# Current Advances in Preclinical Patient-Derived Cultivation Models for Individualized Drug Response Prediction in Pancreatic Cancer

**DOI:** 10.32604/or.2026.075028

**Published:** 2026-04-22

**Authors:** Benjamin Heckelmann, Jannis Duhn, Rüdiger Braun

**Affiliations:** Department of Surgery, University Medical Center Schleswig-Holstein, Campus Lübeck, Ratzeburger Allee 160, Lübeck, Germany

**Keywords:** Pancreatic cancer, precision oncology, patient-derived xenografts, patient-derived organoids, organotypic slice cultures, cancer-on-chip

## Abstract

Pancreatic ductal adenocarcinoma (PDAC) is currently the third leading cancer-related cause of death worldwide and is forecasted to become the second leading cause in the United States by 2030. Despite the development of multimodal treatment regimens, 5-year overall survival remained as low as 12%. Several efforts have been made to account for different aspects of heterogeneous tumor biology in PDAC, aiming to enable treatment stratification of defined subtypes. Besides targeting specific mutations, the definition of molecular (transcriptional) subtypes has gained substantial interest regarding response prediction and treatment stratification. Despite numerous advances in the field of genomic, transcriptomic, and proteomic characterization, the identified biomarkers do not yet facilitate predicting treatment response sufficiently in patients *in vivo*. Considering the growing evidence on the impact of the tumor microenvironment (TME) and intratumoral heterogeneity (ITH) on treatment resistance, there is an unmet clinical need for preclinical cultivation models that allow for predicting treatment response based on individual biological criteria. This review discusses the current advances in such *in vivo* (patient-derived xenografts) and *ex vivo* (organoids, organotypic slice cultures, cancer-on-chip) models for treatment response prediction and stratification in PDAC, and their potential implications in clinical translation.

## 1 Introduction

Pancreatic ductal adenocarcinoma (PDAC) is currently the third leading cancer-related cause of death worldwide and is forecasted to become the second leading cause in the United States by 2030 [1,2]. Once having been diagnosed, radical surgical resection followed by conventional chemotherapy remains the only curative treatment-option for PDAC patients. However, due to long asymptomatic or unspecific courses, only about 10%–15% of the patients are diagnosed in an early stage, amenable to primary resection [2,3].

In recent years, the introduction of polychemotherapy regimens, such as modified FOLFIRINOX (5-fluorouracil, leucovorin, irinotecan, and oxaliplatin), improved the survival of patients from 6.8 to 11.1 months in the metastatic setting and from 35 to 54.4 months in the adjuvant resected setting, compared to gemcitabine monotherapy in large phase-III studies [4,5]. However, despite the introduction of these regimens into clinical guidelines, 5-year overall survival remained as low as 12% [2]. The reasons for the limited survival of pancreatic cancer patients are diverse. A recent cross-validation study of national German and American cancer registries has shown that only about 54% of all patients achieved the goal of guideline-conform therapy, so-called textbook outcome [6]. The selection of (neo)adjuvant treatment regimens, mFOLFIRINOX or gemcitabine (with nab-paclitaxel), is mainly based on the patient’s performance status and comorbidities, whereas the individual tumor biology is currently not considered. Targeted therapies based on individual mutational alterations for (metastatic) PDAC patients did not become an integral part of clinical routine practice [7]. Several efforts have been made to account for different aspects of heterogeneous tumor biology in PDAC, aiming to enable treatment stratification of defined subtypes, which will be outlined in the following sections.

In light of the limited efficacy of chemotherapy in PDAC and mounting evidence for the role of the tumor microenvironment (TME) and intratumoral heterogeneity (ITH) in treatment resistance, there is a clear unmet clinical need for patient-derived cultivation models capable of predicting treatment response based on individual biological features [8,9]. High drug attrition rates in clinical trials are mainly attributed to the use of insufficient and highly artificial preclinical models, neglecting the complexity of the TME [10]. Especially primary cell cultures, which traditionally have been a major tool for drug development and understanding of PDAC biology, seem insufficient, as they neglect the TME [11]. Patient-derived xenograft (PDX) models, established through implantation of patient tumor specimens into immunodeficient mice, are invaluable for bridging preclinical *in vitro* research and *in vivo* studies [12]. Nevertheless, a major limitation of PDX models is their reduced capacity to accurately mimic the human TME [11,13]. In recent years, substantial efforts have led to the development of multiple advanced models that explicitly integrate the TME. Cultivation models such as organoids, organotypic slice cultures, and cancer-on-chip models that reflect multiple tissue components simultaneously are of particular importance. However, each of these models has specific advantages and disadvantages. This review first outlines critical features of PDAC biology with respect to drug response, including the mutational landscape, ITH, and TME. Subsequently, recent developments in *in vivo* and *ex vivo* models that enable prediction and stratification of treatment response are delineated and discussed, focusing on their prospective impact on clinical practice.

## 2 Key Determinants of Treatment Response in PDAC Biology

### 2.1 The Mutational Landscape of PDAC

Over the last decade, large-scale studies using genomic and transcriptomic approaches have made significant progress in understanding the pathophysiology of PDAC [14,15]. It is now widely accepted that the vast majority of PDAC tumors arise from intraepithelial precursor lesions (PanINs), that undergo malignant transformation by accumulation of different mutations in oncogenes and tumor-suppressor genes [16,17]. Most commonly, mutations in the v-Ki-ras2 Kirsten rat sarcoma viral oncogene homolog (*Kras*) initiate neoplastic degeneration of pancreatic ductal epithelium [18,19]. Further, loss-of-function mutations in tumor suppressor genes, e.g., *Tp53*, *Cdkn2a* and *Smad4*, occur during malignant transformation of PanINs [18,20].

As *Kras* mutations are almost ubiquitous in PDAC, great efforts have been made to develop *Kras inhibitors* [14]. Recently, a *Kras*^G12C^ inhibitor has been approved by the FDA for the treatment of advanced non-small-cell lung carcinoma [21,22]. However, this mutation only occurs in about 1.6% of PDAC cases, and a small clinical trial showed only transient responses in a minority of these patients [23,24]. Inhibitors targeting other *Kras*-mutations, such as *Kras*^*G*12*D*^, which are far more abundant in PDAC, are currently being evaluated in clinical trials [25]. In addition, defective DNA repair, i.e., germline and somatic mutations in the *Brca1/2* and *Palp2* genes, has been associated with positive responses to PARP-inhibition and platinum-based chemotherapy [26–28].

The emergence of targeted therapeutics highlights the relevance of molecular profiling for individualized response prediction and therapy selection in (advanced) PDAC [14]. However, only a small fraction of patients harbor driver mutations that are targetable so far. Besides targeting specific mutations, the definition of molecular subtypes has gained substantial interest regarding response prediction and treatment stratification [29].

Collisson et al. introduced three distinct molecular subtypes, termed “quasi-mesenchymal”, “classical”, and “exocrine”, based on transcriptional profiles [30]. Complementarily, Bailey et al. defined four molecular subtypes based on integrated genomic analysis and RNA expression profiles [31]. These findings were complemented by Waddell et al. in 2015, who found differences in genomic (in)stability. Waddell et al. introduced another distinction into four molecular subtypes (stable, scattered, unstable, and locally rearranged), which partially overlapped with the previously proposed subtypes [28]. Recent studies employing large-scale single-cell and spatial transcriptomic profiling uncovered novel heterogeneity within distinct stromal cells, contributing to pancreatic cancer progression [32–34].

In this regard, molecular classification is a promising tool for treatment stratification. In the COMPASS trial, patients were stratified prospectively into defined genomic subcohorts, using Whole Genome Sequencing (WGS) and RNA-sequencing (RNAseq). Patients with a classical subtype had a significantly better response to mFOLFIRINOX compared to those with the basal-like subtype [35]. Currently, the ESPAC-6 trial is prospectively evaluating treatment stratification for adjuvant chemotherapy based on transcriptomic profiling. However, a uniform molecular classification system of PDAC still has to be developed. Although the results to date have only led to informed choice of treatment options in study settings, the collection of molecular data provides a basis for the development of new, specific treatment options and more refined patient stratification.

### 2.2 Intra-Tumor Heterogeneity and Drug Resistance

Whereas the introduced molecular classification systems aim to account for patient-individual tumor differences (inter-tumor heterogeneity), heterogeneity within individual tumors (intra-tumor heterogeneity, ITH) also limits the efficacy of chemotherapy in PDAC [36], as observed in other tumor entities [37]. The underlying reasons for ITH are diverse and have been reviewed by Evan et al. in detail [36]. In pancreatic cancer, cancer-associated fibroblasts (CAFs) were shown to be major contributors to ITH, promoting tumor progression and chemoresistance [38,39]. Cancer stem cells (CSCs) may represent a niche of resistant subclones [40,41], gaining a survival advantage upon chemotherapy and leading to treatment failure. In addition, epigenetic regulation, leading to transcriptomic heterogeneity, can affect the chemosensitivity, and targeting certain epigenetic regulators showed synergistic effects with chemotherapy [42,43].

Recently, our group was able to isolate and characterize gemcitabine-resistant subclones within single-cell-derived PDAC cell lines, reinforcing the relevance of ITH on chemosensitivity. In this study, Gemcitabine-resistant subclones were sensitive towards the inhibition of the epigenetic regulator BET [44].

These results underline the relevance of inter- and intra-tumor heterogeneity for sufficient treatment stratification of PDAC patients. Despite these findings, current clinical guidelines suggest the selection of the chemotherapy regimen based on the performance status (e.g., ECOG scale) of the patient, regardless of the underlying tumor biology.

### 2.3 Importance of the Tumor Microenvironment on Drug Sensitivity

Despite numerous advances in the field of genomic, transcriptomic, and proteomic characterization, the identified biomarkers do not yet facilitate predicting treatment response in the tissue context.

The tumor microenvironment (TME) describes the surroundings of the tumor cells, including proteins building the extracellular matrix (ECM), biomolecules, such as growth factors and cytokines, vessels, and a variety of other cells, especially fibroblasts and immune cells. In PDAC, tumor cells often constitute the minority of cells within the TME. The TME plays a pivotal role in the regulation of tumor growth and metastatic spread [14].

A major component of the TME are CAFs, driving the desmoplastic reaction by deposition of the extracellular matrix [45]. CAFs can be differentiated into subtypes, each contributing differently to the development of PDAC [46,47]. The regulation of CAFs and their diverse impact on PDAC development has been reviewed in detail by Zhang and colleagues [48]. Importantly, CAFs can directly mediate chemoresistance against Gemcitabine by the release of exosomes [49] and alteration of the drug metabolism [50,51].

Recent studies showed a close crosstalk between CAFs and the immune microenvironment. Importantly, CAFs contribute significantly to the immunosuppression in pancreatic cancer. For example, transforming growth factor-β (TGF-β) drives LRRC15^+^ CAFs, which are associated with impaired activity of intratumoral CD8^+^ effector T cell (T_eff_) function and ultimately unfavorable response to immune-checkpoint blockade [52,53].

T_effs_ are key tumor-suppressive cells within the immune-TME by secretion of Interferon-γ (IFN-γ), Tumor Necrosis Factor (TNF), and cytotoxic molecules, directly acting on tumor cells [54]. In pancreatic cancer, a high abundance of tumor-infiltrating T_effs_ positively correlates with patients’ survival [55]. However, PDAC is generally characterized by an immunosuppressive phenotype, mediated by an interplay of CAFs, as well as a predominant infiltration of immunosuppressive M2 macrophages, regulatory T cells (T_regs_), and myeloid-derived suppressor cells (MDSCs), among others [56,57]. The complex mechanisms of the immune microenvironment, as well as novel immunotherapeutic approaches to overcome this has been extensively reviewed [57–59].

Immunotherapy, such as immune checkpoint blockade, has revolutionized the treatment of other solid malignancies over the past few years [60], showed so far unsatisfactory results in PDAC, due to the rare occurrence of microsatellite-instability and scarce infiltration of anti-tumorigenic immune cells [57,61]. Therefore, evaluating patients’ individual immune microenvironment is essential for the success of immunotherapy in PDAC.

Accounting for the complex interplay between fibroblasts, ECM, and immune cells, as well as ITH, Grünwald et al. introduced the concept of subTMEs [38]. The authors showed distinct clusters of “deserted subTMEs”, characterized by ECM-rich matrix and only a paucity of infiltrating immune cells, associated with chemoprotection. In contrast, the “reactive subTME” is characterized by enriched CAFs alongside a high abundance of immune cells, associated with a more aggressive “basal-like” tumor phenotype, but also responsiveness to chemo- and immunotherapy. Interestingly, a transitory “intermediate subTME” exists, and subTMEs’ phenotypes can change during chemotherapy [38]. These novel insights further emphasize that detailed and eventually spatial assessment of patient-individual (sub)TMEs is vital to predict responsiveness towards chemotherapy and thereby tailor precision-oncology approaches in pancreatic cancer.

## 3 Patient-Derived Xenografts

### 3.1 Establishment & Cultivation

**Figure 1:**
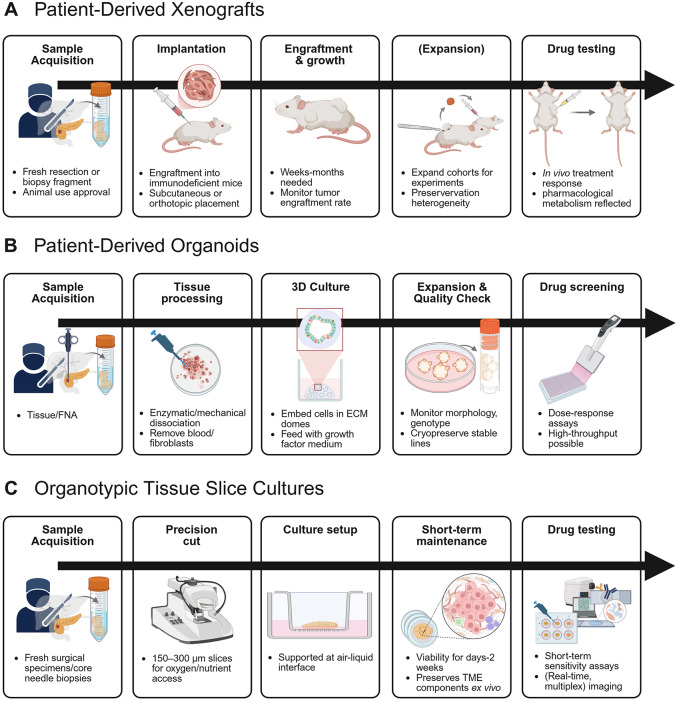

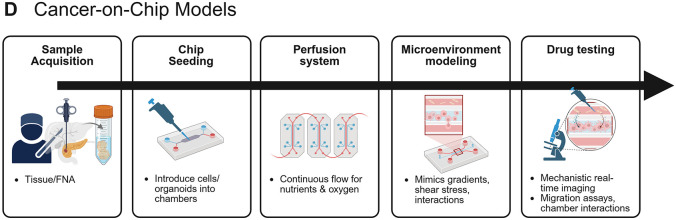
Comparative workflows of patient-derived PDAC culture systems. Experimental workflows and major procedural steps for the principal patient-derived pancreatic cancer model systems: (**A**) patient-derived xenografts (PDXs): tumor fragments from surgical resections or biopsies are implanted into immunodeficient mice. After engraftment and expansion in cohorts, tumors are used for *in vivo* drug testing. (**B**) Patient-derived organoids (PDOs): tumor or fine needle aspiration (FNA) specimens are enzymatically dissociated and embedded in extracellular-matrix domes for 3D culture, expansion, and quality control before (high-throughput) drug screening. (**C**) Organotypic tissue slice cultures (OTSCs): precision-cut tissue slices (150–300 μm) from fresh surgical specimens are maintained *ex vivo* at an air-liquid interface. The native tumor microenvironment is preserved for short-term functional drug testing assays. (**D**) Cancer-on-chip models: versatile culture platforms, patient-derived cells or organoids are introduced into microfluidic chips with continuous perfusion to mimic oxygen and nutrient gradients, enabling real-time imaging and mechanistic drug-response studies. Figure designed with biorender, adapted from the article “*In vitro* models for studying implant-associated biofilms—A review from the perspective of bioengineering 3D microenvironments” by Cometta et al. [67]. Created in BioRender. Heckelmann B. (2026) https://BioRender.com/2knlqy3. ECM: extracellular matrix; TME: tumor microenvironment; FNA: fine needle aspiration.

Animal models are common sources for the translation of preclinical *in vitro* data. Patient-derived xenograft (PDX) models are established mostly by implanting either tumor specimens or cellular components heterotopically or orthotopically into immunocompromised mouse strains (Fig. 1A). More severely immunodeficient mouse strains, such as NOD/SCID or NSG mice, show higher engraftment rates and are more commonly used [12].

Depending on the location of implantation, the PDX tumor mimics the human tumor to a great extent by being attached to the blood flow, taking advantage of the host’s environment. Some highly advanced models offer a solid platform for drug research, and preserve histological characteristics, heterogeneity, genomic and transcriptomic expression profiles over several passages to a certain extent [62–65]. Gao et al. established a tumor-entity overlapping 1075 PDX model comprehensive data bank (n = 42 from PDAC) to generate a validated basis for the clinical translation of PDX-generated data for the use of new anti-cancer drugs. Comparison of the PDX tumor database with The Cancer Genome Atlas (TCGA) and Cancer Cell Line Encyclopedia (CCLE) databases revealed high correlations in mutation rates, particularly between PDX and patient tumors (R = 0.94) [66].

### 3.2 Drug Response & Clinical Translation

PDX models of PDAC have been used for the preclinical validation of numerous standard and investigational drug compounds. In addition to standard drugs such as gemcitabine, cisplatin, nab-paclitaxel, and irinotecan, the effect of more targeted drugs, e.g., directed towards transcription factors, was investigated [12,64,68–73].

Several research groups have used PDX models to predict patient response to therapy. Following the success of adding nab-paclitaxel to the standard drug gemcitabine through preclinical testing in PDX mice, Hidalgo’s group demonstrated the capacity of PDX models to predict treatment response of PDAC patients [73]. In a pilot study in 2011, the authors established 14 PDX mice from different tumor entities, four of them PDAC, and administered a personalized systemic therapy out of 63 options to the respective patient based on the PDX treatment response. One of the four patients showed a response with an exceptional overall survival of 50+ months, as predicted by a tumor growth inhibition rate of 80%. Three additional patients received gemcitabine, which resulted in stabilization of the cancer for two of them; however, one patient still had progressive disease [74]. Izumchenko et al. showed that PDAC-PDX mice adequately predict clinical response to Mitomycin C and Gemcitabine + Paclitaxel in 88% of all clinical cases tested (14 out of 16 patients) based on modified RECIST scores [75]. Stossel et al. established 25 PDX and correlated their response to PARP inhibitors (PARPi) to study the subcohort of germline *BRCA*-mutated PDAC patients for resistance mechanisms. They used organotypic tissue slice cultures derived from the PDXs to characterize sensitivity to platinum agents and PARPi for 27 patients. Their system predicted patient responses with 89% specificity (17/19) and 100% sensitivity (8/8) [76].

### 3.3 Immunotherapy & Limitations

Despite the many advantages of PDXs, there are several limiting factors impairing patient-specific response prediction in PDAC. A major limitation is the reduced mimicry of the human TME. Although the PDX maintains the architecture of the original tumor with respect to typical desmoplastic elements, murine stromal and vascular elements are incorporated into the expanding PDX tumors [11,13,77]. Considering the highlighted importance of the stromal component, this complicates the evaluation of results obtained with PDX models. Another major limitation is the lack of a natural immune compartment. Although PDXs play a critical role in the preclinical evaluation of therapeutic strategies in PDAC, they cannot recapitulate the response to immunotherapeutic agents due to their immunodeficiency. To overcome this limitation, a strategy is to establish humanized PDX models by injecting peripheral blood mononuclear cells (PBMC), engrafting CD34+ cells from donor bone marrow, or implanting CD34+ human hematopoietic stem cells (HSCs) [12,78]. This enables the reconstitution of a human immune system in mice, facilitating research on immunotherapies.

Different methods of humanization of PDX models for various cancer types, their potential implications for immunotherapy research, and limitations have been reviewed in detail [79]. Rosewell et al. showed a local and systemic anti-tumor efficiency of combining chimeric antigen receptor T-cell therapy (CAR-T) directed against human epidermal growth factor-2 (HER-2) in a humanized PDAC-PDX model, proving the feasibility of these models for immunotherapy evaluation in pancreatic cancer [80]. Stossel et al. reported a significant attenuation of the tumor growth rate due to immune checkpoint blockade in humanized glBRCA2-mutated PDAC-PDX with high mutational load [76]. Likewise, dendritic cell-based immunotherapy evoked systemic immune responses followed by tumor regression and prolonged survival in HSC-humanized mice [81]. However, these strategies are limited by graft versus host effects (GvHD) and the difficulty of obtaining HSCs from cancer patients [12,78,82].

The predominant model with intact TME and most frequently used for evaluation of immunotherapeutic strategies is genetically engineered mouse models (GEMMs) harboring pancreas-specific expression of mutant KRAS. The most frequently used KrasLSL-G12D/+, Trp53LSL-R172H/+, Pdx1-Cre (KPC) mice model was exploited for the investigation of combination strategies of immune checkpoint inhibitors and treatments targeting several TME components, such as the stromal [83] and myeloid compartment [84,85]. However, the KPC model is preclinical and not patient-derived by definition. Despite the high value for precision oncology approaches, GEMMs and humanized PDX models are mostly limited to being preclinical models primarily for drug development and understanding of potential biomarkers. Therefore, results are usually only indirectly applicable in routine clinical practice and personalized medicine.

Another limiting factor using PDX models for individual patient-specific treatment stratification is the preselection of tumor samples due to engraftment bias. Garrido-Laguna et al. reported an engraftment rate of 61%, Jun et al. of 55.8%, and Stossel et al. of 54% in PDX models of pancreatic cancer. These groups reported an association of engraftment with tumor size, site of resection, stromal pathway enrichment, metastatic gene expression signature, and poor prognosis. About 25% to 30% of implants fail even under optimal conditions [74,76,86,87]. Another critical challenge for the use of PDX models as a preclinical therapy prediction tool is the establishment time of about 6–8 months [74,88].

While humanized mouse models are feasible for mechanistic validation and prioritization of combination strategies, they are time-consuming and resource-intensive, making them unsuitable for use as a ‘real-time avatar’ for individual treatment decisions. In addition, their use is associated with ethical considerations, including animal welfare concerns, high animal numbers required for statistically meaningful experiments, and repeated invasive procedures, which collectively limit scalability and routine clinical translation.

## 4 Patient-Derived Organoids

### 4.1 Establishment & Cultivation

3D organoid cultures have been developed in addition to 2D cell cultures and PDX. Huch et al. initially described the isolation of pancreatic ducts after enzymatic digestion of healthy pancreatic tissue (Fig. 1B). Manually retrieved pancreatic ducts were subsequently seeded in growth-factor reduced Matrigel and cultivated for several days to weeks [89]. Boj et al. adapted this protocol for the cultivation of murine and human neoplastic pancreatic tissue. Subsequent orthotopic transplantation of patient-derived organoids (PDO) into WT mice led to the development of invasive PDAC remaining a regular ductal architecture [90]. PDO cultures have also been established for a variety of other cancer types, e.g., rectal, prostate, bladder, ovarian, and lung cancer [91–95]. PDOs maintain genomic and metabolic heterogeneity over the culture period and show extensive ITH [96–99]. In addition, integration of CAFs into PDO cultures enables modeling of tumor-stroma interactions [100,101].

Tiriac et al. were the first to generate PDOs from endoscopic ultrasound (EUS) guided fine-needle biopsy samples, and successfully isolated PDOs in 87% from the tumor specimens [102]. However, 66% reached the 5th passage of growth. The method was later validated by other groups [103–106]. More recently, PDOs together with fibroblast cultures were generated from a single-pass fine needle biopsy [107]. Grützmeier et al. showed successful implementation of PDO-CAF co-cultures in 19.2% of EUS-guided biopsies. Co-cultivation with CAFs increased growth rates and viability [108].

### 4.2 Drug Response & Clinical Translation

PDAC-PDOs showed differential response following treatment with epigenetic regulators, which was linked to conservation of phenotypic heterogeneity after cultivation. This study highlighted the potential of PDOs for drug sensitivity assays [109]. Further, cultivation of PDOs with conditioned media from certain CAF subtypes resulted in increased gemcitabine-resistance. This indicates that PDOs remain fully reactive and can recapitulate chemosensitivity in a context-dependent manner [38]. Retrospective analyses showed a high predictive value of PDOs for clinical treatment response to conventional chemotherapy [110,111]. Prospectively designed trials validated the predictive value of PDOs for conventional chemotherapy responses [112,113]. This is in line with trials conducted in other tumor entities, e.g., Xu et al. recently reported that the predictive accuracy of employing PDO in rectal cancer can reach up to 93.75%, depending on the time of testing [114].

In the study of Beutel et al., the predictive value was 91% for first-line drugs in treatment-naive patients and declined to 40% in pretreated patients [112]. In line, Demyan et al. reported an accurate prediction in 71% of PDOs derived from neoadjuvant-treated tumors, likely due to the increasing importance of TME and immune-mediated resistance mechanisms that are not adequately modeled in mostly epithelial PDOs [115,116]. However, recently, a study conducted by Boilève et al. reported a sensitivity of 83.3% and specificity of 92.9% in 87 pretreated patients [117]. Apoptotic responses and tumor-stroma cell proportions following *ex vivo* treatment of PDOs can be quantified using a 3D immunofluorescence assay, potentially adding to the predictive value of PDO-based drug sensitivity analyses [118].

The establishment of PDOs from fine-needle biopsies yields high potential for treatment response prediction for neoadjuvant therapy, where larger tissue specimens for cultivation are not available. In this regard, Demyan et al. were able to show that biopsy-derived PDOs can predict responses to neoadjuvant chemotherapy within 7 days from tissue sampling in a rapid screening assay [115]. Likewise, Oyama et al. were able to predict Gemcitabine-resistance using PDOs derived from fine-needle biopsies [119]. From a translational perspective, the predictive accuracy of PDO drug response for clinical outcomes was observed to be high for first-line chemotherapy, especially in treatment-naive patients [110].

Clinical correlation evidence supporting PDO-guided functional precision oncology in PDAC has expanded in recent years [120]. Feasibility studies have evaluated the integration of PDO establishment, and several phase II and III clinical trials (e.g., NCT04931394, NCT04931381) are currently recruiting or are ongoing to implement PDO-drug testing into existing clinical workflows [120]. Another recent study investigated the use of phenotypic PDO drug screening as part of a phase III clinical trial evaluating the use of comprehensive precision medicine in PDAC [121]. The utilization of PDAC-PDOs for personalized therapy has previously been reviewed in detail by Bengtsson et al. [122] and Beutel et al. [120]. In summary, PDO models of PDAC provide a promising platform for drug sensitivity screening, especially regarding the possibility of obtaining results from fine needle biopsy samples, which might enable therapy prediction before starting neoadjuvant therapy.

### 4.3 Immunotherapy

Several groups have successfully established PDO/immune cell (mostly PBMC) co-cultures to model tumor-immune interactions and shifts in T cell subtypes [123–126], mimicking the immunosuppressive TME in PDAC. Emerging multicomponent approaches additionally incorporate stromal elements, e.g., CAFs, together with PBMCs to recapitulate immune exclusion and stromal barrier characteristics [127]. Depletion of myeloid-derived suppressor cells together with immune-checkpoint blockade with the PD-1 antibody Nivolumab restored T cell immunity and induced tumor regression [128]. Likewise, *ex vivo* treatment with anti-PD-1 antibody Avelumab and anti-HER2 antibody Trastuzumab induced Natural Killer (NK) cell-induced apoptosis of PDAC-derived organoid/immune cell cultures [129]. Beyond PBMC co-cultures, Air-liquid interface (ALI) cultures can retain immune and stroma cells, including T cells, B cells, NK cells, and macrophages, and preserve the original T cell receptor spectrum, successfully modelling immune checkpoint blockade [126]. Based on these findings, PDOs might, to some extent, be suitable to predict responses towards immunotherapeutic agents. To our knowledge, no studies have yet compared *ex vivo* response towards immunotherapy with clinical data to further validate this method.

### 4.4 Limitations & Challenges

A key limitation of conventional PDO systems is the loss of the native TME during tissue dissociation and culture establishment. Conventional PDOs primarily consist of epithelial cells and lack a stromal and immune compartment, which hinders the recapitulation of TME-dependent drug response mechanisms. Considering the well-established roles of CAFs, ECM, and myeloid-dominated immune suppression in mediating chemoresistance and immunotherapy failure, the reductionist cellular composition of PDOs inherently limits their capacity to model TME-dependent resistance mechanisms [84,130,131]. In addition, PDO generation relies on enzymatic tissue dissociation, which disrupts spatial tissue organization and eliminates physical interactions between tumor cells, stromal barriers, and tumor-infiltrating lymphocytes. As a result, critical *in vivo* features such as gradients of oxygen, cytokines, and drug penetration are not preserved, potentially leading to an overestimation of drug sensitivity *in vitro* [110,111].

These limitations are reflected in the reduced predictive performance of PDOs in the neoadjuvant setting, as exemplified by Demyan et al., where prior treatment exposure diminishes concordance between organoid responses and clinical outcomes. This effect is even more pronounced in PDOs derived from tumors treated with neoadjuvant gemcitabine plus nab-paclitaxel, a regimen known to exert substantial effects on the stromal compartment [115,116]. Together, these observations suggest that the absence of stromal and immune-mediated resistance mechanisms in conventional PDO cultures contributes to the declining predictive accuracy observed in pretreated tumors.

Another important challenge in PDO-based drug response prediction is the presence of synergistic and antagonistic treatment interactions, which can reduce predictive accuracy if not explicitly assessed, as recently demonstrated by Xu et al. in PDOs from rectal cancer [114]. However, it appears that, in contrast to chemoradiation therapy protocols in colorectal cancer, interaction effects among standard chemotherapies in PDAC, such as FOLFIRINOX or gemcitabine plus nab-paclitaxel, are generally modest [132]. Consequently, multi-agent combination screening may offer limited additional predictive value over single-agent pharmacotyping for FOLFIRINOX- or gemcitabine/nab-paclitaxel-based treatment selection.

Establishment of PDOs requires technical expertise, a well-equipped laboratory, and can be relatively time-consuming, especially when co-cultures with CAFs are required. Differences in tissue processing, culture media composition, passaging strategies, and drug response readouts contribute to variability across laboratories, complicating data comparability. Especially, immunotherapy assays are incompletely standardized, also due to their higher technical complexity. However, in comparison to other patient-derived models, protocols for PDOs are established, and ongoing clinical trials aim to assess standardization and automation across multiple trial sites [120].

## 5 Organotypic Slice Cultures

### 5.1 Establishment

A cultivation approach that avoids disintegration and subsequent assembly is the establishment of organotypic slice cultures (OTSCs) (Fig. 1C). The cultivation methods for OTSCs are heavily influenced and optimized by neuroscience research [133]. In particular, the advantage of tissue culture on a semipermeable membrane was initially described by a neuroscience research group [134]. Around the 1990s, this *ex vivo* method was transferred to cancer research and was established for a variety of tumor entities, including liver, prostate, lung, head and neck, colorectal, gastric, and pancreatic cancer [135,136]. OTSCs were also established for non-cancerous pancreatic tissue, e.g., to investigate the role of cell-cell interactions in pancreatic tissue from diabetic patients [137–142]. Using this method, fresh tumor tissue, mostly from surgical resections but also core needle biopsies, is processed into sections between 200 and 500 μm thickness and cultivated on semi-porous polytetrafluoroethylene (PTFE) membranes to ensure optimal oxygenation and nutrient supply [143–146]. The newer vibratome has an advantage over the earlier Krumdieck Tissue Puncher, since the combination of cutting and vibrating motion of the blade seems to cause less tissue damage [147,148]. By bypassing the disintegration of the tissue, the focus of this culture system is on successful cultivation and preservation of the component ratios—instead of establishing a culture from individual (epithelial) tumor clones. By preservation of the stromal cell population, the extracellular matrix, as well as the distinct immune cell populations, this model is sought to mirror the original tumor with exclusion of a functioning vasculature. For the cultivation of the various cell types of a PDAC specimen, the use of basal cultivation media such as DMEM or RPMI Media, in addition to the optional use of trypsin inhibitors for enzyme digestion protection, was reported by several groups [144,146,149].

### 5.2 Cultivation

OTSCs of PDAC are viable for a limited number of days. Although minimal necrotic and apoptotic lesions were observed already after 24 h, the overall histological and cytological features and grade of differentiation were retained up to 96 h [149]. Lim et al. observed a gradual decrease in viability during a cultivation time of 9 days, indicated by histopathological viability scores and immunohistochemical expression of the proliferation marker Ki67 and apoptosis marker Cleaved Caspase 3 (cC3). However, gross morphology did not change over the duration of culture [145].

There were no significant changes in the main constituting cell populations forming the TME, confirming preservation of the tissue heterogeneity. The consistent expression of immune- and stromal markers supports the suitability of OTSCs to investigate tumor-stroma-immune interactions [145,149]. In line with these immunohistochemical results, a proteomic analysis using liquid-chromatography/mass-spectrometry (LC/MS-MS) by Jiang et al. revealed that most immunologic proteins remained stable over six days of culture [150].

Profiling of the genome-wide transcriptome of 15 OTSCs from 5 distinct tumor specimens revealed no differentially expressed isoforms at any time point from day 0 to day 3, supporting transcriptional stability of OTSCs. However, Ghaderi et al. observed an upregulation of 15 and downregulation of 25 genes, partially associated with apoptosis and cell death, which is in line with the histological correlates [149,151]. Furthermore, using genome-wide transcriptome sequencing analysis, Szekerczés et al. identified significant upregulation of seven genes after treatment of seven distinct OTSC specimens with indole-3-pyruvic acid (IPA). IPA is an agonist of the aryl hydrocarbon receptor (AhR) signaling pathway, which reduces oxidative cell damage and has tumor-suppressive properties. By inducing *Cyp1a1*, *Cypb1*, *Ahrr*, and *Tiparp* genes—all associated with the AhR signaling pathway—they further validated the reflection of accurately preserved cell biology in patient-derived OTSCs on a transcriptomic level [152]. Therefore, critical biologic pathways seem to remain intact in OTSCs, therefore enabling the assessment of response towards modern precision medicine approaches beyond cytotoxic chemotherapies.

### 5.3 Drug Response

OTSCs allow for the investigation of treatment effects in a preserved tissue context [153]. In proof-of-principle experiments with cycloheximide and kinase-inhibitor staurosporine, time-dependent and dose-dependent treatment effects could be measured by histological (measurement of necrotic areas) and immunohistochemical analysis (Ki67 and cC3 expression). To evaluate metabolic activity, phosphorylation of ribosomal protein S6 was compared between treated and untreated OTSCs. A strong expression of phosphorylated S6 (pS6) indicated high metabolic activity of OTSCs 24 h after establishment. Treatment with the mTOR inhibitor rapamycin led to a substantial reduction in pS6 expression [149].

In initial experiments with clinically relevant chemotherapeutic agents such as gemcitabine and cisplatin, histopathological analyses revealed heterogeneous tissue responses. Gemcitabine, for instance, led to a marked inhibition of proliferation, whereas cisplatin did not induce a comparable effect [145].

Focusing on treatment response, Moro et al. treated OTSCs with various concentrations of sodium selenite. By establishing a viability score, partially based on neoadjuvant regression grading parameters for PDAC, they observed a significant decrease in PDAC viability while preserving non-neoplastic tissues. Validated by whole transcriptome sequencing, which showed a decrease of growth- and metastasis-associated genes, Moro et al. suggested sodium selenite to be a promising treatment option due to its tumor-specific cytotoxicity in OTSCs [154].

### 5.4 Immunotherapy

Both Seo et al. and Rohila et al. exploited the preservation of the immune cell population in human PDAC OTSCs, focusing on tumor-infiltrating CD8^+^ T cells. Seo et al. combined OTSCs, T cell receptor (TCR) sequencing, and flow cytometry to analyze the effects of immunotherapeutic agents on anti-tumor activity within the PDAC TME. Increased tumor cell death was accompanied by lymphocyte expansion after treatment of OTSCs with a combined PD-1- and CXCR4-inhibition. Furthermore, live microscopy showed that CD8^+^ T cells were migrating from normal stroma areas into the juxtatumoral compartment, accompanied by an increase in tumor cell apoptosis [155]. Rohila et al. demonstrated the complementarity of genetically engineered mouse models (GEMMs) and OTSCs to understand the role of the macrophage spleen tyrosine kinase (Syk) for the promotion of PDAC growth and metastasis in GEMMs. Furthermore, Syk-inhibitor Fostamatinib (R788) remodeled the suppressed immune microenvironment of gemcitabine-treated orthotopic mouse models and OTSCs. More precisely, R788 shifted protumorigenic macrophages towards an immunostimulatory phenotype and boosted the antitumorigenic response of CD8^+^ T cells, ultimately leading to tumor regression [156].

### 5.5 Clinical Translation

Although Rohila et al. validated their findings by observing similar results in both human *ex vivo* PDAC OTSCs and orthotopic mice models [156], a study using human *ex vivo* PDAC OTSCs as an instrument to predict and stratify treatment is still lacking, yet regarding the high clinical demand.

### 5.6 Limitations & Challenges

A principal limitation of OTSCs is the limitation to short-term cultivation. Although the overall tissue architecture and cellular composition are preserved for several days, gradual loss of viability and increased apoptotic activity occur with prolonged cultivation [145,150]. While preserving endogenous immune cells, the recapitulated immune compartment is static and progressively exhausted over time. The continuous recruitment of immune cells, the priming of lymphocytes in secondary lymphoid organs, and systemic immunomodulation cannot be modeled. Therefore, OTSCs are well-suited for studying short-term immune-tumor interactions and immune checkpoint-dependent mechanisms, but they cannot capture adaptive immune responses, memory formation, or systemic toxicities caused by immunotherapies [155,156].

From a technical standpoint, preparing an OTSC requires high-quality, fresh tissue; precise slicing; and standardized handling. Variability in slice thickness, tissue composition, and culture conditions can significantly impact viability and treatment response [154]. This variability can lead to inconsistencies between laboratories and limit scalability compared to more standardized systems, such as organoids. Compared with organoid cultures, OTSCs also generally require greater tissue input, which can constrain their use in patients for whom only limited biopsy material can be obtained.

Although OTSCs enable the functional evaluation of treatment-induced phenotypic alterations within an intact tissue context, achieving quantitative and reproducible response metrics remains challenging [136]. Current readouts rely largely on labor-intensive and partially subjective methods. The integration of automated, high-content imaging and spatial molecular analyses to enable standardized, large-scale screening is still under active development.

## 6 Cancer-on-Chip and Microfluidic Devices

### 6.1 Establishment & Cultivation

The potentially most advanced 3D cell culture models are often referred to as “cancer-on-chip” cultures (Fig. 1D). This term refers to a heterogeneous group of biotechnological constructs whose main characteristic is to mimic the TME as closely as possible. In addition to modeling different tissue components, this often refers to the supply of oxygen and nutrients via the culture medium. Due to the presence of heterogeneous cell populations, these 3D cell cultures are particularly sensitive to variations in culture conditions. The supply of oxygen and nutrients by diffusion through the tissue is often limited. To overcome this hurdle, many cancer-on-chip models focus on precise delivery and control of the culture medium flow using valves, minipumps, and chemical gradients. Other important components of the newer cancer-on-chip models are materials that simulate an extracellular matrix, particularly hydrogels, i.e., soft materials formed by biopolymers, cellular fibers, and mini capsules. A guaranteed supply of culture medium components through these microfluidic-based applications could enable the establishment of long-term cultures and overcome the limitations of diffusion-based nutrient supply [157].

### 6.2 Drug Response

Haque et al. combined patient-derived organoid cultures and a 3D-printed microfluidic device consisting of two distinct chambers separated by a semi-porous membrane. One of the chambers housed the organoid cultures, while the other supplied the cell constructs with RPMI medium by a continuous flow. After a culture period of 26 days, the cancer cells were positive for phosphorylated ERK, which the authors interpreted as an adequate cell survival. To prove the functional unity of their cancer-on-chip model, the response to gemcitabine alone was compared with gemcitabine plus anti-stroma agents (all-trans-retinoic acid and liposomal clodronate). The stroma-depleting agents resulted in a twofold higher apoptosis rate, as measured by cC3 expression, compared to gemcitabine monotherapy [158].

### 6.3 Clinical Translation

Steinberg et al. combined spheroid cultures of various tumor specimens with a 3D-printed microfluidic device for multiple drug screening assays. The viability and spheroid area of ten *ex vivo* spheroid cultures, including three from PDAC and one from a colorectal metastasis of a pancreatic neuroendocrine adenocarcinoma, correlated positively with the patients’ clinical response to the chemotherapeutics oxaliplatin, gemcitabine, etoposide, and mitomycin, but in parts inversely for 5-FU, cisplatin, and bevacizumab. Although one of the *ex vivo* spheroid cultures accurately reflected the clinical response of the corresponding patient, the study primarily addressed the challenges of identifying reliable treatment response indicators rather than establishing a robust clinical correlation. The authors note, a key challenge was that spheroids occasionally increased in size even with escalating concentrations of cytotoxic agents. Steinberg et al. conclude that size-based evaluation should be analyzed together with other functional assays to draw accurate conclusions. This is consistent with the ubiquitous need for accurate readout systems for *ex vivo* 3D culture systems [159].

### 6.4 Limitations & Challenges

Cancer-on-chip methods usually rely upon established patient-derived models, such as PDOs or OTSCs, which are leveraged by more advanced technical approaches, such as microfluidic devices. Therefore, many biological limitations are inherited, including incomplete immune representation and the absence of systemic host factors. Furthermore, one could interpret the investigation of Cancer-on-chip culture systems as a way of tackling certain limitations of the above-discussed model systems. For example, Hughes et al. linked OTSCs with a continuous perfusion of medium, to optimize cell growth and metabolism in the slices, which was indicated by an increased lactate accumulation and proliferation capacity measured by Cyclin D1 [160].

While microfluidic perfusion can partially overcome diffusion-related constraints and improve tissue viability, these platforms remain highly customized with respect to chip design, flow parameters, extracellular matrix composition, and analytical endpoints, resulting in limited reproducibility across laboratories [161]. A key limitation in cancer-on-chip models is the standardization across labs due to high technical complexity. Appropriate read-outs are still investigated and are often non-validated, e.g., spheroid size and metabolic activity, differ between publications, and may not reflect therapeutic efficacy reliably [159]. High cost and limited throughput restrict their integration into time-sensitive clinical workflows. To date, clinical validation of cancer-on-chip models is limited, and most studies have focused on providing concept and mechanistic insights rather than establishing robust correlations with patient outcomes, although for other tumor entities, such as melanoma and appendiceal cancer, feasibility trials have been accomplished [162,163].

At this point, cancer-on-chip systems are an encouraging experimental development for patient-derived models, but they have not yet become a clinically proven platform for predicting individualized therapy in PDAC [164].

## 7 Conclusion & Outlook

Recently, the AVATAR trial prospectively evaluated standard therapy based on physicians’ discretion vs. precision medicine following whole exome sequencing and patient avatars utilizing PDX or PDO models. Only a minority of patients (10.2%) in the experimental arm received personalized treatment. Overall, the use of avatar systems was not associated with improved survival. This underscores the difficulty of implementing personalized precision medicine approaches in patients with pancreatic ductal adenocarcinoma (PDAC), who often experience rapid clinical deterioration before receiving personalized medicine. However, in the subcohort of patients receiving matched therapy, survival was improved [121]. Thus, the study highlights the potential of avatar models while also pointing out the need to integrate them more effectively into clinical settings. Further clinical trials, especially evaluating PDO-guided selection of chemotherapy, are currently recruiting (NCT04931394, NCT04931381). Likewise, the ESPAC-6 trial aims to compare selection of adjuvant chemotherapy by transcriptomic signature vs. standard selection based on clinical decision making, including a large PDO biobank for correlative analyses (NCT05314998), reflecting the current, yet unmet clinical need for integration of predictive methods towards precision medicine in pancreatic cancer.

The patient-derived culture systems discussed here offer a broad and complementary spectrum to cultivate tumor specimens and test individual patients’ treatment response. Each model has its strengths and weaknesses, which also depend on the available laboratory infrastructure. In addition, reduction and refinement in animal research are major aims; *ex vivo* models are favorable for implementation in clinical routines. In addition, economic considerations are also important to enable the broad availability of these methods, apart from academic high-volume centers, with PDO and OTSC models being comparably cheap and easy to implement in comparison to PDX or Cancer-on-Chip models (Table 1). Additionally, recently developed and pre-clinically tested implantable microdevices might enable *in vivo* drug response prediction, especially in patients ineligible for surgical resection [165–167]. However, to our knowledge, there are yet no studies evaluating implantable microdevices in pancreatic cancer.

**Table 1:** Comparison of patient-derived culture systems for personalized drug testing.

Model	Maintenance	TME Preservation	Studying of Interactions	Throughput	Time to Response Readout
**PDX**	High	High	Limited	Low	Long
	Long-term animal care and monitoring	Retains *in vivo* tumor architecture, but mouse stroma replaces human	Mouse stroma and lack of human immune cells constrain analysis	Limited by animal numbers and long growth time	Months until measurable tumor growth
**PDO**	Medium	Medium	Medium	High	Short
	Requires specialized medium and sterile handling	Epithelial component preserved, Stroma and immune limited unless co-cultured Engineered assembly	Possible via co-culture with fibroblasts or immune cells	Suitable for 96-/384-well screening formats	Drug testing is feasible within weeks of culture establishment
**OTSCs**	Low	Very high	High	Low	Very short
	Short-term culture (days to approx. week)	Maintains patient stroma, ECM, immune cells in preserved tissue architecture	Endogenous cell-cell and matrix interactions maintained	Limited number of slices per tissue specimen	Treatment response measurable within days
**Cancer-on-Chip**	High	High	Very high	Medium	Short
	Requires microengineering setup and flow maintenance	Recapitulates microenvironment under controlled flow conditions Engineered assembly	Allows defined multi-cellular co-cultures and dynamic interactions	Microfluidic multiplexing allows parallel testing	Real-time or within days depending on setup

Note: PDX: Patient-derived xenograft; PDO: Patient-derived organoid; OTSCs: Organotypic tissue slice culture.

From our perspective reflected in this overview, there is no perfect *ex vivo* culture system that, on its own, can accurately predict an individual patient’s treatment response. Depending on the application, single or multiple models should be applied in parallel. Key points to obtain the most accurate results in preclinical research and personalized medicine that can be translated to clinical practice are the preservation of molecular profiles, including ITH, and the histological context, including tumor microenvironment (TME). PDX models remain important, particularly for preclinical drug discovery, because molecular profiles and histology can be obtained over multiple passages and correlate well with those of the original tumors. However, organoids and OTSCs offer a promising alternative that overcomes engraftment bias and allows for deeper evaluation of tissue components such as stromal, ECM, and immune components.

**Figure 2:**
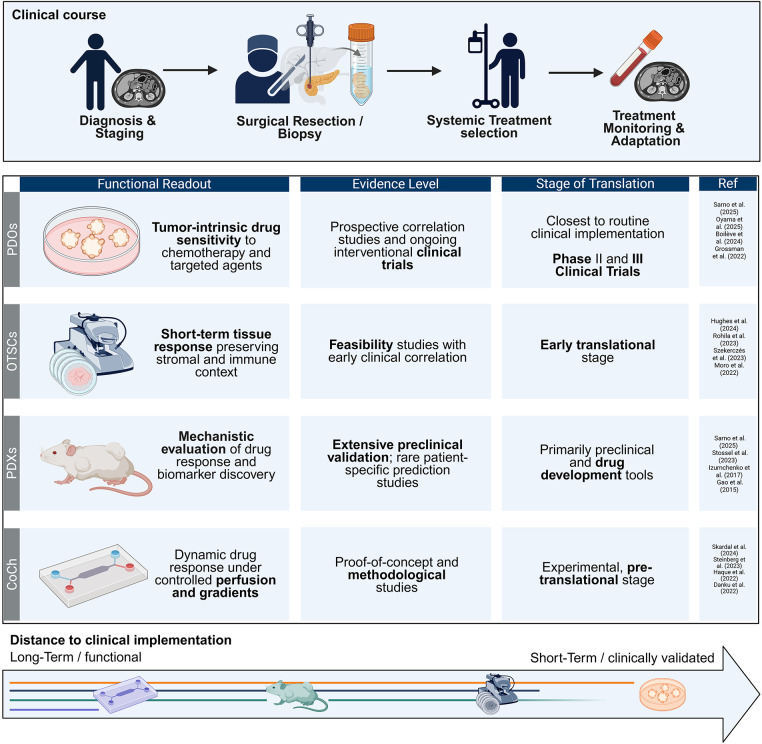
Integration of culture systems towards clinical practice. Hierarchical integration of patient-derived culture systems for precision oncology and clinical translation. Patient-derived xenografts and cancer-on-chip platforms are positioned on the functional end of the spectrum, supporting mechanistic interrogation and early drug development. Patient-derived organoids provide a scalable, near–real-time option for therapy stratification and functional precision medicine within clinically actionable timelines. Organotypic tissue slice cultures and explant models occupy an intermediate position by preserving key tumor microenvironment components for short-term response testing, but are constrained by higher tissue demand and limited throughput. Created in BioRender. Heckelmann B. (2026) https://BioRender.com/da0w2sw. PDOs: patient-derived organoid; OTSCs: organotypic tissue slice culture; PDXs: patient-derived xenografts; CoCh: cancer-on-chip [66,75,76,113,117,119,121,152,154,156,158–162].

To make progress in the field of therapeutic stratification, in addition to efforts to create the most accurate models that simulate the original tumor *in vivo* as closely as possible, it is necessary to ensure correlation through prospective studies. Personalized *ex vivo* treatment response prediction approaches require models that can be established quickly, are easily scalable, and yet are close enough to the donor tissue to predict the therapeutic effect without major biases. Even if PDOs are manipulated by prior disintegration, they can be easily scaled, and the influence of different tissue components, including the immune component, can be studied. Although PDOs are tissue signatures rather than exact replicas of donor tissue, they were shown to accurately predict treatment response [122]. Further developments of cancer-on-chip models are promising to advance current 3D (co-)cultivation methods of PDOs. Particularly for the evaluation of novel compounds within preclinical or personalized clinical frameworks, accounting for the TME is crucial, as exemplified by immune checkpoint blockade therapies. In this context, OTSCs have several advantages compared to PDOs due to the preservation of donor stromal and immune components. Another advantage of OTSCs is their rapid establishment. However, the cultivation period is strongly limited to several days. Both methods, PDOs and OTSCs, can potentially be improved by the implementation of cancer-on-chip technologies. Table 1 summarizes the advantages and disadvantages, whereas Fig. 2 outlines the progress of the discussed patient-derived models towards integration into clinical routine practice.

Ultimately, the combination of the emerging field of spatial OMICs and *ex vivo* cultures will provide links between phenotypic, transcriptomic, and proteomic drug response. Tissue-specific drug response can be assessed alongside spatial molecular analyses to provide personalized insights and can complement non-spatial OMICs approaches. Considering and ideally preserving the spatial arrangement of the various tissue components of primary cancer will enable assessing the effects of drug compounds at the molecular level and in the context of the TME [99,168]. OTSCs and multicellular PDOs offer the potential to discover the role of subpopulations of tissue components, shedding more light on stromal and immune tissue patterns, and their influence on drug distribution and processing. In addition, correlations between the phenotypic and transcriptomic response of *ex vivo* culture systems and the primary tissue samples could enable the identification of signatures in the tissue of origin. These might facilitate therapy stratification, based on already established histopathologic diagnostics [169]. Linking the multiple levels of the individual molecular markup will likely provide new insights to understand mechanisms of drug response and resistance in complex multicellular tumor tissues such as pancreatic cancers. The implementation of bioinformatics expertise in patient-derived models, e.g., by creating histological classifications based on machine learning or deep learning, is essential for understanding the influence of the TME and ITH on treatment responses [170,171]. Results based on patient-derived models should be decoded as accurately as possible, and linked to conventional histological and OMICs information to allow indirect clinical translation of the collected research data [172]. While each patient-derived model presents distinct advantages and limitations, the combined use of multiple systems enables a more holistic investigation of individual treatment response. With the continued advancement of precision oncology, patient-derived cultivation models are becoming increasingly important for informing individualized treatment approaches.

## Data Availability

The authors confirm that the data supporting the findings of this study are available within the article.
